# Targeting Potential of Innate Lymphoid Cells in Melanoma and Other Cancers

**DOI:** 10.3390/pharmaceutics15072001

**Published:** 2023-07-21

**Authors:** Hobin Seo, Amisha Verma, Megan Kinzel, Qiutong Huang, Douglas J. Mahoney, Nicolas Jacquelot

**Affiliations:** 1 Department of Biochemistry and Molecular Biology, Cumming School of Medicine, University of Calgary, Calgary, AB T2N 4N1, Canada; hobin.seo@ucalgary.ca (H.S.); megan.kinzel1@ucalgary.ca (M.K.); 2 Department of Microbiology, Immunology and Infectious Diseases, Cumming School of Medicine, University of Calgary, Calgary, AB T2N 4N1, Canada; djmahone@ucalgary.ca; 3 Arnie Charbonneau Cancer Research Institute, Calgary, AB T2N 4N1, Canada; 4 Department of Biological Sciences, University of Calgary, Calgary, AB T2N 4N1, Canada; amisha.verma1@ucalgary.ca; 5 The University of Queensland Frazer Institute, University of Queensland, Woolloongabba, QLD 4102, Australia; qiutong.huang@uq.edu.au; 6 Walter and Eliza Hall Institute of Medical Research, Parkville, VIC 3052, Australia

**Keywords:** cancer, cytokines, immune cells, immune checkpoints, immunotherapy, innate immunity, innate lymphoid cells, melanoma, NK cells, PD-1

## Abstract

Reinvigorating the killing function of tumor-infiltrating immune cells through the targeting of regulatory molecules expressed on lymphocytes has markedly improved the prognosis of cancer patients, particularly in melanoma. While initially thought to solely strengthen adaptive T lymphocyte anti-tumor activity, recent investigations suggest that other immune cell subsets, particularly tissue-resident innate lymphoid cells (ILCs), may benefit from immunotherapy treatment. Here, we describe the recent findings showing immune checkpoint expression on tissue-resident and tumor-infiltrating ILCs and how their effector function is modulated by checkpoint blockade-based therapies in cancer. We discuss the therapeutic potential of ILCs beyond the classical PD-1 and CTLA-4 regulatory molecules, exploring other possibilities to manipulate ILC effector function to further impede tumor growth and quench disease progression.

## 1. Introduction

Cancer treatments have traditionally focused on targeting the cancer cells themselves, to destroy malignant tumors. While efficient in some cases, the vast majority of patients do not benefit from these therapies. Moreover, many suffer from adverse events due to off-target effects on healthy cells [1,2]. Over the past three decades, with discoveries of the cellular and molecular basis of immunity in cancer and various technological advancements, we progressively observed a paradigm shift in cancer treatment with a change in focus—eliminating cancer cells not through direct targeting but by reinvigorating the patient’s own immune system. The development of these so-called “immunotherapies”, designed to restore immune cell activity against tumors and to overcome tumor-induced suppression of such responses, has revolutionized our approach of treating cancer patients, showing striking improvement in disease prognosis. One approach consists of blocking the function of certain molecules responsible for restraining the immune responses of T cells, best known as negative regulators or “immune checkpoints”. Based on the spectacular response rates observed in clinical trials, specific antibodies targeting cytotoxic T lymphocyte-associated protein 4 (CTLA-4), programmed cell death 1 (PDCD1, known as PD-1), and more recently, lymphocyte-activation gene 3 (LAG-3) signaling have been approved for the treatment of various tumor types, including melanoma [3,4,5,6,7]. The management of metastatic cancer has been revolutionized by these immune checkpoint blockers (ICB), particularly in advanced melanoma, which has seen significantly improved survival rates and better tumor control [8,9]. However, a number of cancer patients remain refractory to these treatments, owing to various mechanisms that allow tumors to resist treatment [10]. In addition, treatments with ICB are often associated with mild to severe toxicities [11], ranging from cutaneous toxicities [12] and colitis [13], to myocarditis [14]. Therefore, considerable efforts have been deployed to better understand the mechanism of action of ICBs and to identify potential biomarkers of clinical utility [10]. As T cells are not autonomous and rely on innate cells for their responses, expanding into targeting immune cells other than T cells for immunotherapy has become an intensive area of research. This review will focus on the family of innate lymphoid cells (ILCs) and the recent literature studying their function, role, and targeting potential in cancer with respect to ICB. We will discuss novel ILC-based immunotherapies, extending beyond immune checkpoint inhibition. These include cytokine-based targeting, understanding the tumor microenvironment (TME)-mediated plasticity of ILC phenotypes, ILC engineering, and different vectors to direct ILC activity. These approaches highlight the possible clinical applications of targeting these unique cell populations.

## 2. Characteristics of ILCs

**Figure 1:**
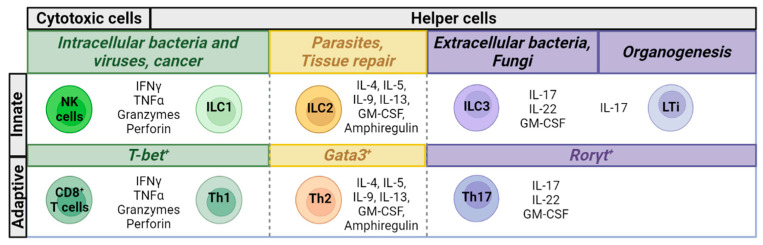
Transcription factors, effector molecule expression, and cellular activities of adaptive and innate lymphoid cells.

The ILC family is composed of five subsets: natural killer (NK) cells, type 1, 2, and 3 ILCs (ILC1, ILC2, and ILC3, respectively) and lymphoid tissue inducer (LTi) cells (Figure 1). Derived from the common lymphoid progenitor, these cells differ from adaptive lymphocytes in that they do not express antigen-specific receptors and are rather activated by the integration of cytokines, hormones, neurotransmitters, and various stress and microbial signals from their environment [15,16]. Therefore, ILCs respond earlier to immune threats than T cells, influencing subsequent adaptive immune responses. With similar transcriptional profiles to their T cell counterparts, NK cells mirror the phenotype and effector functions of cytotoxic CD8^+^ T cells while ILC1, ILC2, and ILC3 types reflect the signature profiles of T helper-1 (Th1), Th2, and Th17 CD4^+^ subsets, respectively [17] (Figure 1). NK cells and ILC1s that express the transcription factor Tbet and secrete interferon-gamma (IFN-γ) mediate type 1 immunity toward intracellular pathogens, viruses, and tumors. ILC2s produce cytokines in line with type 2 immune responses toward parasites and allergens and express the transcription factor GATA3 in higher frequencies than other subsets. ILC3s are enriched in the gastrointestinal tract where they respond to extracellular bacteria and fungi, are characterized by their transcription factor RORγt and can produce IL-17A and IL-22. LTi cells also rely on RORγt for their development and survival and are involved in secondary lymphoid tissue formation during embryonic development [18]. These qualities broadly characterize ILCs, but each subset exhibits certain levels of both heterogeneity and plasticity based on the changes in their environment, which can result in transdifferentiation from one subset to another [19]. NK cells are primarily found in circulation, whereas type 1–3 ILCs, also referred to as helper ILCs, reside in tissues, particularly in peripheral barrier sites [20,21]. Albeit at low frequencies, helper ILCs can also be detected in human peripheral blood by flow cytometry [22] and constitute under 0.2% of all peripheral blood mononuclear cells [23]. In humans, 5–20% of lymphocytes in circulation are NK cells [24].

ILCs play important roles in tissue homeostasis and host immunity against external pathogens [25,26]. More recently, they have been implicated in cancer, influencing anti-tumor responses, treatment efficacy, and disease outcomes [27].

## 3. ILCs and the Immune Response to Tumors

ILCs have been shown to accumulate in mouse and human tumor tissues and to exhibit various activated profiles [28]. These tumor-infiltrating ILC subsets drive both pro- or anti-tumor immunity, depending on contexts which have been comprehensively summarized elsewhere [27,29,30]. However, considerable progress over the past few years has been made in the cellular and molecular pathways responsible for these seemingly paradoxical activities.

Unlike their CD8^+^ counterparts, NK cells directly target and kill cancer cells that show little to no MHC class I expression [31]. NK cells are strongly associated with better treatment response and lower disease severity in hematological malignancies and can also control primary solid tumor growth and metastasis [32]. In melanoma, we and others found that NK cells infiltrate both primary tumors and melanoma metastases, and high expression of an NK cell gene signature was associated with improved overall survival [33,34,35,36]. However, several studies have also identified impaired and dysfunctional NK cell populations correlating with poor prognosis in human hepatocellular carcinoma [37], esophageal squamous cell carcinoma [38], high risk B- and T-cell acute lymphoblastic leukemia (ALL) [39], and acute myeloid leukemia [40]. More recently, ILCs with immunosuppressive capacities harboring an NK cell-like phenotype were identified [41]. In ex vivo tumor-infiltrating cultures from high-grade serous ovarian carcinoma samples, Crome and colleagues found that CD56^+^ ILCs suppressed autologous T cell expansion in an NKp46-dependent manner [41]. In breast cancer patients, high expression of the ecto-nucleotidase CD73 on NK cells correlated with larger tumor size [42]. Mechanistically, CD73^+^ NK cells were associated with increased IL-10 production, suppressing CD4^+^ T cell proliferation and IFN-γ production [42]. Although NK cells have long been studied for their anti-tumor activity, these investigations demonstrate that NK cells have the capacity to contribute to cancer progression.

Although perforin and granzyme-mediated cytotoxicity in ILCs is a characteristic often assigned to NK cells, certain ILC1 populations also present cytotoxic capability [32]. For instance, tumor-resident ILC1s in chromophobe renal cell carcinoma express granzyme A in response to IL-15 derived from cancer cells [43], and other mouse models have shown granzyme-expressing ILC1s [32,44]. Nixon et al. identified ILC1 subsets expressing granzyme B, perforin, and granzyme C in the liver and salivary glands of mice [45]. They further found that granzyme C-expressing ILC1s expanded in mammary tumors and can mediate anti-tumor responses, a mechanism dependent on transforming growth factor beta (TGF-β) and IL-15 signaling [45]. Further anti-cancer function of ILC1s can arise from their production of IFN-γ, and these ILC1s can stunt the progression of leukemia stem cells in vivo [46]. While IFN-γ can act in the TME to restrict tumor growth, induce apoptotic pathways, and stimulate other immune cells to increase anti-tumor activity, IFN-γ can also exhibit pro-tumorigenic functions at sustained or low doses, and low IFN-γ levels are correlated with poor prognosis in non-small cell lung cancer (NSCLC) [47,48,49]. Additionally, regulating feedback mechanisms of IFN-γ can lead to the expression of inhibitory ligands, including PD-L1, contributing to immune suppression [47]. In melanoma, Ercolano et al. found that the peripheral blood and tumor-infiltrated lymph nodes (TILN) of patients were enriched for ILC1s as compared to healthy donors [50]. These ILC1s had impaired IFN-γ production, and investigations indicated that this suppression is influenced by metabolites kynurenines and adenosine in the TME [50]. In pre-clinical mouse models, the cytokine TGF-β has been reported to mediate immune evasion in the TME through the conversion of NK cells into ILC1 and intermediate ILC1 populations [51,52]. The current data on ILC1s suggest that transdifferentiated ILC1 subsets display more pro-tumor roles [32]. Overall, there is a key role in cytokines produced by the TME as a determinant of ILC1 function, particularly IL-15 and TGF-β [29,43,45].

ILC2s also act on both sides of tumor immunity. They have been shown to promote pro-tumor myeloid-derived suppressor cell function in acute pro-myelocytic leukemia [53], *Apc*-driven colorectal cancer (CRC) [54], and bladder cancer [55]. Under hypoxia, ILC2s can transdifferentiate into an IL-10^+^ ILC immunosuppressive phenotype similar to that of regulatory T cells and are implicated with tumor progression in the hypoxic environments of pancreatic ductal adenocarcinoma (PDAC) [56]. ILC2s respond to thymic stromal lymphopoietin (TSLP), IL-18, IL-25, and IL-33, of which the latter two garner interest due to their roles in stimulating various ILC2 activities in cancer ([18,57] p. 10)). In the *Apc*-driven mouse models of CRC, IL-25-stimulated ILC2s are responsible for directing a tumor-promoting microenvironment [54]. A recent study found that although IL-33 mediated innate anti-tumor responses dependent on NK cell activity, IL-33 also activated ILC2s which in turn suppressed NK cell function, possibly through CD73 expression [58]. In lung melanoma metastases, IL-33-activated lung ILC2s are key drivers of disease progression [59]. Mechanistically, ILC2-derived IL-5 production induces eosinophil-mediated suppression of the anti-tumor NK cell function [59]. In contrast, in primary melanomas [60,61] and in PDAC [62], the IL-33-ILC2 axis was associated with anti-tumor responses, further highlighting the complexity of this subset and the TME influencing these responses. In ILC2-driven responses, eosinophils infiltrate melanoma lesions to control tumor progression [60,61]. However, the lactic acid produced by melanoma cells suppresses ILC2 proliferation, survival, and IL-5 production [61], leading to reduced eosinophil infiltration and increased tumor growth. Several studies also correlate ILC2s to positive outcomes in CRC [63,64], in which one points to anti-tumor CD8^+^ T activation through ILC2-produced IL-9 [64].

Human ILC3s can be further divided into two groups based on their expression of NKp44 or NKp46, defined as natural cytotoxicity receptor negative (NCR^−^) or NCR^+^ ILC3s [29]. Although defined as a helper population, ILC3s from both the blood and tissue that have been co-cultured with cells of different cancers have exhibited cytotoxic capabilities, mediated by a TRAIL-TRAILR2 pathway [65]. With the continuous expression of RORγt, these ILC3s show a cytotoxic phenotype unique from NK cells and ILC1s [65]. Whether these cells represent canonical ILC3s or a NK cell/ILC3-like subset with specific developmental trajectories and particular effector functions remains to be determined. However, under specific stimulation, ILC3s transdifferentiated into an ILC1/NK cell-like subset bearing cytotoxic features may emerge that are associated with anti-tumor responses in preclinical mouse models [66]. More recently, Bruchard and colleagues observed ILC3 activation and their recruitment into tumors upon cisplatin treatment in mice [67]. Activated ILC3s produce the chemokine CXCL10 which mediates the recruitment of T cells to the tumor, promoting anti-tumor immunity and increasing the efficacy of ICB treatments [67]. In CRC, reduced ILC3s in human tumors or the ablation of MHC Class II expression on ILC3s in mice are both associated with poor disease outcomes [68]. Importantly, specific impairment of this ILC3-CD4^+^ T cell dialogue through MHC class II deletion induces immunotherapy resistances in mouse models. ILC3s also constitutively secrete the cytokine IL-22, particularly in the intestine, where its accumulation promotes tissue barrier integrity and epithelial cell renewal and proliferation [69]. In the context of tumors, however, IL-22 overexpression has been associated with tumor growth and poor disease outcomes [69,70].

Collectively, these studies report that ILCs mediate both pro- and anti-tumorigenic function, depending on the tumor type and the organ involved. In melanoma for instance, we have described the association between NK cell gene signatures and survival, impaired ILC1 function mediated by the TME, and ILC2-driven anti-tumor responses. However, similarly activated but in a different environment, lung ILC2s support the progression of lung melanoma metastases. These differences highlight the specificities that exist between tissues and suggest that pro- or anti-tumorigenic ILC function depends on the disease stage. Our current understanding of ILCs in melanoma development and progression remains limited, warranting further investigation to determine how ILCs mediate skin homeostasis and protect against melanoma development—likely through interacting with cells in their environment [71]—to realize their targeting potential [72]. Overall, there is a strong need to better understand (i) the diversity within ILC subsets, including their propensity to transdifferentiate into other ILC subsets in tumors, and (ii) the critical role of tissue-derived signals in driving ILC-specific responses, to efficiently target these populations.

## 4. ILCs and Immune Checkpoint Molecules

**Figure 2:**
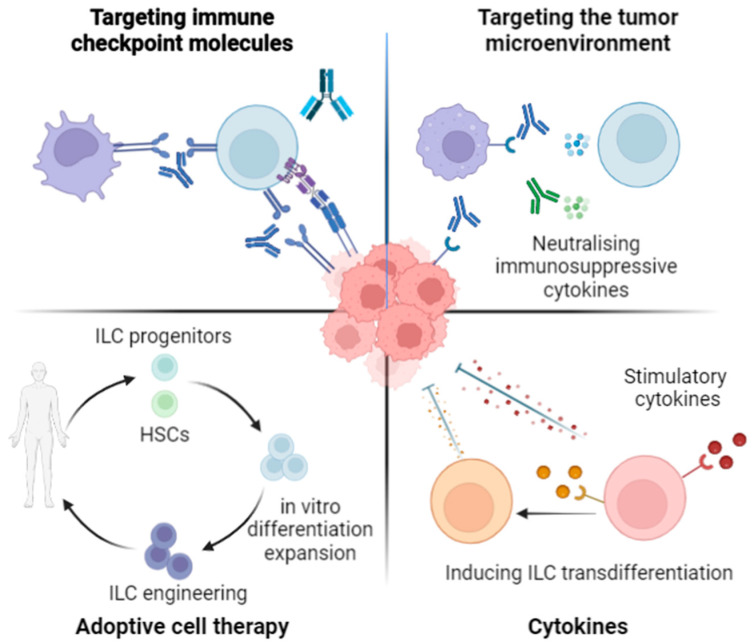
Targeting approaches as to manipulate ILC effector function in cancer.

Immune checkpoint molecules limit the inflammatory immune responses that are rapidly elicited following the detection of a danger signal. Indeed, if left uncontrolled, inflammatory responses may induce significant tissue damage, organ failure, or even worse, lead to patient death. Tissue-resident ILCs constitutively express many immune checkpoint molecules and like adaptive lymphocytes, tumor-infiltrating ILCs express inhibitory molecules which mostly dampen their effector functions [73]. Although ILCs express diverse immune checkpoint molecules, which are briefly discussed in this section, little is known about how currently approved immunotherapies (Table 1) impact immune checkpoint-expressing ILCs and whether ILC subsets participate in the efficacy of ICB. Detailed expression patterns of these molecules according to ILC subsets have been comprehensively reviewed in other sources [73,74,75,76]. Here, we provide an overview on the latest observations describing the expression patterns of PD-1 on ILCs as well as NK-specific inhibitory molecules and their targeting potential (Figure 2).

### 4.1. PD-1 Expression Patterns

Amongst all the inhibitory molecules expressed by ILCs, PD-1 is probably the best studied. Originally identified on T cells in 1992 [96], PD-1 expression has since been described on many immune cell types, including ILCs and their progenitors (Table 2). ILC bone marrow progenitors (Lin^−^CD127^+^Flt3^−^α4β7^+^c-kit^+^) expressing PD-1 give rise mainly to non-NK ILCs when adoptively transferred into Rag2^−/−^γc^−/−^ mice [97,98], thus identifying PD-1 as a marker to confidently isolate ILC progenitors. However, no defect in the generation of mature ILCs has been reported in *Pdcd1*^−/−^ mice compared to wildtype controls [98,99], raising questions on the role and function of this receptor during ILC development.

Mouse ILC1 express low levels of PD-1 at steady-state [97]. However, in tumors, an ILC1-like subset that expresses ILC1 markers, but which mostly results from the transdifferentiation of NK cells under TGF-β signaling, expresses intermediate levels of this inhibitory receptor [51]. In humans, ILC1 cells that infiltrate breast and gastrointestinal tumors also express high levels of PD-1 [28]. Therefore, PD-1 expression on ILC1s seems to be induced upon inflammation, such as on cells infiltrating tumors. In many studies, PD-1 expression has been detected on mouse and human NK cells, particularly on tumor-infiltrating populations [28,100,101,102,103,104,105,106,107,108]. A recent report, however, suggests that NK cells fail to express PD-1 endogenously [109]. In this context, Hasim and colleagues recently uncovered that NK cells had the ability to acquire PD-1 expression on their membrane through trogocytosis [110], a process involving the exchange of parts of the cellular membrane during the interface between two cells. Thus, is it possible to reconcile both observations.

In the lung, 20–40% of ILC2s express PD-1, of which the expression increases following influenza infection or challenge with papain allergen [97]. PD-1 expression can also be induced on ILC2s by IL-33 and γc cytokines [60,62,99,111,112]. With regards to its function, PD-1 negatively regulates ILC2 activity, particularly the KLRG1^+^ subset, blocking ILC2 proliferation by inhibiting the STAT5 function [99]. In the absence of PD-1 signaling, the inflammatory KLRG1^+^ ILC2 subset increases in frequency and function [99]. Furthermore, ILC2 proliferation is metabolically regulated by PD-1; metabolic shifts toward glycolysis, glutaminolysis, and methionine catabolism are observed in PD-1-deficient ILC2s, with increases in GATA-3 and Ki67, a key proliferation marker [111]. Observed by utilizing humanized mouse models of asthma, proper regulation through PD-1 is important in ameliorating ILC2-mediated airway hyperreactivity [111]. PD-1 is therefore an important negative regulator of ILC2 effector function [99,111,113,114,115,116]. We and several other groups found that tumor-infiltrating ILC2s (TILC2s) express PD-1 [28,60,62,117,118,119]. These PD-1^+^ TILC2s have been implicated in tumor progression in CRC [117], as well as in NSCLC, in which PD-1^hi^ TILC2s exhibit immunosuppressive qualities [119]. These PD-1^hi^ TILC2s produce higher levels of IL-4 and IL-13 than PD-1^low^ TILC2s, leading to enhanced M2-like macrophage polarization in vitro [119]. In melanoma [60] and pancreatic ductal adenocarcinomas [62], however, IL-33-induced TILC2s play anti-tumor roles and their anti-tumorigenic functions are further enhanced by the blockade of PD-1.

PD-1^hi^ cells are also precursors to the ILC3 lineage [97], and PD-1^+^ ILC3s are found in mouse lung [97], intestine [120], and decidual tissues [121]. Human ILC3s expressing PD-1 have also been reported in the decidua, and it is suggested that they play important roles in mediating immune tolerance during pregnancy [121]. In intestinal ILC3s, PD-1 signaling regulates the metabolic function of the cells by promoting glycolysis and lipid metabolism, enhancing IL-22 expression [120]. PD-1 deficient intestinal ILC3s have decreased IL-22 expression resulting in increased epithelial damage and loss of barrier integrity during inflammation. In cancers, PD1^+^ ILC3s were reported in breast and gastrointestinal tumors [28]. ILC3s are also the most prevalent ILC subset in the malignant pleural effusions of many different cancers, and the variable expression of PD-1 on these ILC3s is suggested to limit their anti-tumor activity through interactions with its ligand PD-L1 on tumor cells [107].

**Table 2:** Summary of PD-1 Expression on Mature ILC Subsets.

Population	Mouse Expression	Human Expression	Function	References
NK cells	Expressed particularly on tumor infiltrating populations		Negative regulator	[28,100,101,102,103,104,105,106,107,108]
ILC1s	Low level at steady state ILC1-subsets in tumors express intermediate levels	High levels in tumor infiltrating populations Expressed in PBMCs of cancer patients	Potential inhibitory role	[28,51,97]
ILC2s	Expressed in tumor infiltrating populations 20–40% of lung ILC2s, increases upon inflammation Substantially expressed in the colon	Significantly expressed in tumor-infiltrating populations Expressed in PBMCs of cancer patients	Regulates airway hypersensitivity Negative regulator of ILC2 function	[28,60,62,99,111,113,114,115,116,117,118,119]
ILC3s	Expressed in mouse lung, colon, decidual tissues Expressed substantially on LTi cells residing in the colon and in other gut tissues, upregulated upon activation	Expressed in the decidua Expressed in breast and GI tumors Low expression in PBMCs of cancer patients	Mediating immune tolerance during pregnancy Promoting metabolism and maintaining barrier function in the intestine Potential inhibitory role in cancer	[28,97,120,121]

PBMC, peripheral blood mononuclear cell; GI, gastrointestinal.

### 4.2. Immune Checkpoint Targeting Potential of ILCs

Cancer cells evade T cell-mediated killing by downregulating their MHC expression, which then allows NK cells to detect these cells and exert cytotoxic activity [122]. This innate function was first described in the 1980s as the “missing-self hypothesis”, where NK cells were shown to selectively target lymphoma cells with a loss of MHC class I expression [123]. Inhibitory receptors on the surface of NK cells specific for HLA-class I molecules thus mediate a tolerance toward healthy cells and include inhibitory members of the killer cell immunoglobulin-like receptor family (KIRs) and the NKG2A/CD94 heterodimer [124]. While NKG2A is conserved in humans and mice, mice do not express KIRs and rather rely on the functional equivalency of lectin-like Ly49 receptors [125]. It is now evident that these MHC class I-specific receptors play important roles in the functional development of NK cells, known as NK cell education, where NK cells are calibrated to later recognize target cells through the “missing-self” program [126,127,128,129,130]. Of note, although a small population of mature NK cells that do not express at least one of these inhibitory receptors was described, these cells are anergic or hyporesponsive [124,126]. KIRs recognize classical HLA-A, -B, and -C molecules while human NKG2A is specific to the non-classical HLA-E [124], and both have been recent targets in immunotherapy.

Initial preclinical models targeting inhibitory Ly49 receptors and transgenic inhibitory KIRs in mice showed promising data in support of developing anti-KIR antibodies for human trials [131,132,133,134]. However, a phase II trial of the anti-KIR2D antibody identified as IPH2101, administered to patients with smoldering multiple myeloma, showed no clinical efficacy [135]. Upon investigation, it was found that IPH2101 blocked KIR2D but also induced a loss of free KIR2D expression on NK cells, resulting in hyporesponsive function [135]. A recombinant version of this antibody, IPH2102, or lirilumab, has since been studied for its safety and efficacy in different cancers, primarily in combination with other strategies [136,137,138,139,140].

NKG2A is broadly expressed on tumor-infiltrating NK and CD8^+^ T cells in several human tumors, and many of these tumors express HLA-E [141]. Based on these observations, a phase II clinical trial in squamous cell carcinoma of the head and neck patients was initiated (NCT02643550), investigating the combination of a human anti-NKG2A antibody, monalizumab, with the standard of care anti-epidermal growth factor receptor antibody cetuximab. An additional cohort was included to study the triplet of monalizumab, cetuximab, and durvalumab, an anti-PD-L1 agent [142]. The efficacy data from this trial showed promise to the potential of monalizumab-based therapies [141,142]. Other trials have since demonstrated the therapeutic potential of monalizumab in combination with immunotherapies in metastatic microsatellite-stable CRC [143] and NSCLC [144,145]. Durvalumab is the current FDA-approved treatment for patients with stage III unresectable NSCLC with no disease progression following chemoradiation [145,146]. The phase II trial COAST (NCT03822351) demonstrated that patients with unresectable stage III NSCLC receiving a combination of monalizumab and durvalumab respond better than those receiving durvalumab alone [144]. This combination, and the combination of durvalumab plus oleclumab, an anti-CD73 monoclonal antibody, is being investigated further in the phase III trial PACIFIC-9 (NCT05221840) [145].

With the expression of immune checkpoint molecules, and notably PD-1, reported throughout ILC subsets, there is great interest toward targeting ILCs with ICB strategies. Both PD-1 and PD-L1 blockade have shown to induce NK cell anti-tumor function [100,147,148]. Although PD-1 blockade is a known therapy to activate tumor-infiltrating T cells, emerging evidence has shown that TILC2s also benefit from anti-PD-1 antibody, activating their ability to carry out anti-tumor responses [60,62]. In various human cancers, TILC2s have been shown to express PD-1 [28,60,62], demonstrating great interest in targeting ILC2s through the combination of PD-1 blockade to further augment tumor immune responses.

Our group and others have reported the presence of all ILC subsets in the tumors, TILN, and peripheral blood of melanoma patients [50,60,149]. Cristiani et al. reported that following anti-PD-1 therapies in melanoma patients, the percentage of mature CD117^−^ ILC2s was increased in the periphery, with improved TNFα production of CD117^+^ ILCs and IL-13 from CD117^+^ ILC2s [149]. Although PD-1 blockade increased the percentage of CD117^−^ ILC2s, overall survival was correlated to a lower frequency of this subset in the periphery, potentially suggesting that there may be a requirement for the migration of these ILC2s into the tumor for a positive response [149]. In a study using B16 melanoma mouse models, an upregulation of PD-1 on pulmonary ILC2s was found to create a tumor-permissive environment [150]. Similar to the findings of Cristiani et. al, PD-1 blockade increased the production of TNFα by these ILC2s, directly mediating B16 melanoma tumor cell death [150].

Previously, our group has described the importance of ILC2s in melanoma control, and their potential to be targeted by PD-1 blockade. We have shown that the ILC2-derived granulocyte–macrophage colony-stimulating factor (GM-CSF) is crucial for melanoma control through the recruitment of eosinophils [60]. Although the activation of ILC2s after IL-33 stimulation increases GM-CSF production, PD-1 was also found to be upregulated on activated ILC2s [60]. We observed that the combination of IL-33 and anti-PD-1 antibody significantly drives anti-tumor responses toward melanoma by promoting ILC2s and cytotoxic eosinophil infiltration into tumors [60]. As the TILC2-eosinophil axis is correlated with increased survival in melanoma patients, these investigations point to the clinical potential of targeting ILC2s to improve immunotherapy responses toward melanoma.

While the targeting of ILCs using ICB is of great interest, alternative targeting approaches have emerged as to take advantage of their tissue distribution and effector functions to further improve anti-tumor responses (Figure 2).

## 5. Novel ILC Targeting Approaches in Cancer

### 5.1. Targeting Cytokines and Their Receptors

The activation and phenotype of ILCs are heavily dependent on their exposure to specific cytokines in the environment, which indicates the potential to apply certain cytokine-based strategies to enhance ILC anti-tumor function and disease outcomes. The IL-33/ILC2 axis has shown to drive anti-tumor responses in melanoma [60,61,151] and orthotopic pancreatic tumors [62], and we have described the effect of IL-33 in combination with PD-1 blockade to significantly increase the anti-melanoma response [60]. IL-33 has a broad reach and can stimulate NK and CD8^+^ T cells, regulatory, and type 2 CD4^+^ T cells, as well as other innate lymphoid cells, eosinophils, mast cells, and basophils [152]. In addition, IL-33-activated ILC2s have been shown to produce IL-13 [111,153], driving a pro-tumorigenic TME [119]. As described earlier, ILC2s activated with IL-33 can also suppress NK cell function. Further investigations on the role of IL-33 are required, as it varies heavily based on cancer type and the TME composition.

In cancers where the cytokines produced by ILCs drive tumor progression, such as PD-1^hi^ ILC2-derived IL-4 and IL-13 in NSCLC, one strategy would consist of blocking their pro-tumorigenic functions. The ability to target both IL-4 and IL-13 simultaneously is attributed to the expression of the IL-4 receptor α chain (IL-4Rα) subunit in both IL-4 and IL-13 receptors [154]. Dupilumab, a monoclonal antibody targeting IL-4Rα to block both IL-4 and IL-13 signaling, is approved for treating atopic dermatitis and asthma [155] and eosinophilic esophagitis [156] and is a promising treatment against prurigo nodularis [157], another T_H_2-associated disease. The effect of dupilumab on ILC2s has been explored in the context of allergic disorders [158,159] and is an approach that has yet to be investigated in ILC2 cancer immunology.

In melanoma, existing IFN-α and IL-2 targeting monotherapies marked the introduction of cytokine-based immunotherapy but have also highlighted challenges that are facing the field, such as low response rates and high toxicities [160]. The future of cytokine treatments depends on their strong synergistic potential with other approved therapies, improving their concentration and persistence in the TME and the use of various vectors to ensure the specific and accurate localization of the cytokines without systemic inflammation [160].

### 5.2. Influencing ILC Plasticity and Transdifferentiation

ILC subsets are heterogeneous and exhibit a great deal of plasticity during their development and maturation in tissues. In addition, transdifferentiation from one to another subset is exacerbated during inflammation [19]. It is proposed that the balance between alternative ILC lineages is controlled by the network of transcription factors in ILCs [19], further influenced by the signals they receive from their environment. For instance, one recent investigation has characterized a spectrum of subsets within a ILC3 to ILC1 transition, suggested to be mediated at the transcriptional level through Aiolos and T-bet and influenced by the tissue microenvironment [161]. In human colon cancer, tumor-infiltrating ILC3s transition to an ILC1 phenotype, with an expected increased expression of Aiolos transcripts [68]. This dysregulation of ILC3s in the colon has revealed that the interaction with CD4 T cells through MHC class II expression on ILC3s is important for both type 1 immune responses in the TME and reducing resistance to PD-1 blockade [68]. Studies have shown that ILC2s convert into IFN-γ producing ILC1s through IL-12 signals, a mechanism associated with airway inflammation [162] and Crohn’s disease [163]. The ability for ILC2s to undergo a non-canonical pathway and exhibit an ILC3-like phenotype has also been reported, expressing RORγt and producing IL-17 [164,165]. Similarly to ILC3 to ILC1, NK cells to ILC1s is a conversion that exhibits higher levels of plasticity compared to ILC2 transdifferentiation into ILC1s or ILC3s [19]. A recent study profiling tumor-infiltrating ILCs during the progression of a colitis-associated CRC mouse model found different proportions of subsets from early to late stage disease [117]. Wang et al. found that within the tumor, ILC1 frequency was reduced in the late stage and, compared to the early stage, exhibited a suppressed profile, expressing inhibitory receptors and producing less IFN-γ. Out of the three ILC2 subsets identified, the group dominant in the late stage of CRC tumors was PD-1^hi^, compared to early-stage PD-1^low^ ILC2s, and exhibited pro-tumor function. Interestingly, ILC3s were mainly identified in the early stages and during tumor progression transdifferentiated into immunosuppressive ILCs, producing IL-10 to promote tumor progression. They also reported similar frequencies and phenotypes of ILC subsets in analyzed human tissues of advanced CRC, confirming the translational relevance of their work. To introduce the therapeutic potential of targeting the transdifferentiation of ILC3s to immunosuppressive ILCs, Wang and colleagues demonstrated that depletion of this subset or IL-10 deletion suppressed tumor growth significantly and blocking TGF-β signaling through receptor deletion on ILC3s or TGF-β inhibitor treatment blocked the ILC3 to immunosuppressive ILC conversion, also controlling tumor growth. Blocking TGF-β could additionally inhibit the transdifferentiation of NK cells into tumor-promoting ILC1s [51]. While explored in the clinic, the efficacy of anti-TGF-β strategies remains to be demonstrated [166]. Further investigations are warranted to better understand how the TME promotes ILC transdifferentiation toward tumor-promoting function. Such advances would significantly unveil new strategies for ILC targeting.

### 5.3. ILC Differentiation, Expansion, and Engineering: Chimeric Antigen Receptors and TCRs

Chimeric antigen receptor (CAR)-T cells have revolutionized cancer treatment. Independent of MHC receptor signaling, engineered CARs can guide T cells to cancer cells expressing specific antigens, induce T cell activation, and the killing of tumor cells. Based on exceptional clinical activity, CAR-T cells have received FDA approval for the treatment of hematological malignancies [167]. However, the CAR-T cell field still experiences several barriers that limit treatment efficacy. These include antigen specificity, antigen loss, trafficking and infiltration into solid tumors, and immunosuppressive TMEs [167]. As ILC subsets have emerged as important effector cells in anti-tumor responses, one avenue of interest is applying CAR technologies toward ILCs.

Anti-CD19 CARs therapies are well studied, due to the expression of CD19 on many B cell cancers [168]. In 2020, Liu et al. described the potential of applying anti-CD19 CAR constructs onto NK cells. Tested in mouse models of lymphoma, engineered NK cells derived from cord blood, which were transduced with anti-CD19 CAR, IL-15, and inducible caspase 9, showed strong anti-tumor responses [169]. In phase I and II trials with patients of CD19^+^ lymphoid cancers, the CAR-NK cells showed positive responses without the induction of toxic effects associated with CAR-T cell therapy, namely cytokine release syndrome and neurologic toxic effects, although they did observe high-grade transient myelotoxicity [167,170]. One key characteristic of these cord blood NK cells is their allogeneicity. Compared to allogeneic T cells, NK cells do not carry the risk of causing graft-versus-host-disease [171,172], a potentially fatal complication where the infused T cells attack host tissue. Liu et al. further demonstrated the complete tolerance of moderate to full HLA-mismatching in patients [170]. With no requirement to engineer autologous cells as in T cell therapies, along with the potential to mass produce cellular products from one donor, NK therapies provide a quicker and more cost-efficient promise [171]. Enhancing their targeting capacity, inhibiting negative regulation, and increasing survival and persistence are areas in study to improve the viability of allogeneic NK cells as future off-the-shelf treatments [171]. Other reviews have further dived into the exciting field of CAR-NKs and NK-based immunotherapy, discussing the different cell sources, including memory-like and stem cell-derived NK cells, the targeting potential in solid tumors, and how to improve general NK therapies through strategies including cytokine armoring and enhancing tumor trafficking [173,174,175,176]. One drawback of CAR therapies is their restriction to targeting of antigens expressed on the surface of cells. T cell receptor (TCR) based therapies, which allow for the recognition of non-surface antigen, have also been expanded to NK cells [177]; modified TCR-NK cells have been developed, shown to mimic characteristics of T cells while continuing to carry out effector NK functions [178]. TCR-NK cells offer an alternative solution to engineered TCR-T cells, where the lack of endogenously expressed TCR chains on NK cells avoids mispairing issues with heterodimerization [177]. A different study has demonstrated the production of TCR-ILC-like cells, from a T_H_1 clone specific for the HLA-DR9 restricted b3a2 peptide in chronic myelogenous leukemia [179]. From these T cells, they generated induced pluripotent stem cells (iPSCs) and differentiated them into a population of T cells (referred to as iPS-T cells) expressing characteristics of NK/ILC1s, while continuing to express the TCR of the T_H_1 clone. While not expressed on these iPS-T cells, the transduction of CD4 conferred the ability to respond in a TCR-dependent manner. The induction of a specific CD40L^hi^ ILC1-like subset was demonstrated, and the group characterized their ability to promote antigen-specific cytotoxic T lymphocyte responses via the conditioning of dendritic cells.

More recently, Li et al. found that CAR-transduced PSCs were able to preferentially differentiate into T cells or ILCs based on the strength of CAR signaling, establishing for the first time an anti-CD19 CAR ILC2 population following lentiviral transduction of H1 embryonic stem cells [180]. While exhibiting the phenotypic and functional aspects of canonical ILC2s, these H1-CAR ILC2s also expanded when stimulated with CD19^+^ NALM6 cells. Based on the strength, antigen presence, and modulation of CAR signaling, they showed the ability to divert T cell differentiation to an ILC2 lineage. Further, Ueda and colleagues transduced an anti-glypican-3 (GPC3) CAR, targeting GPC3 that is expressed in liver and ovary cancers but rarely in healthy tissue, into iPSCs [181]. They differentiated these CAR-iPSCs through hematopoietic and lymphocyte progenitor states to an iCAR-NK/ILC-like cell product. The manipulation of this iCAR-NK/ILC generation for clinical relevance, their ability to suppress GPC3-expressing tumor growth, and their safety have also been studied by the group and show promising data to support further investigation. Further considerations of their study include how to enhance the CAR-dependent anti-tumor activities of these NK cells and to combine cytokine strategies to further activate their cytotoxic potential.

### 5.4. Bi- and Tri-Specific Vectors

A concept now being explored in NK cells is applying bi- and tri-specific antibodies. These antibodies are specific against either two or three epitopes, recognizing different antigens [182]. Bi-specific T cell engagers (BiTEs) target a tumor antigen together with a T cell surface molecule, often CD3 [182]. Such T cell engagers (TCEs) have emerged as a novel therapy directing the interaction between T cells and cancer cells, leading to TCR-dependent tumor cell killing [183]. Blinatumomab was the first TCE approved by the FDA and has opened the door to the study of TCEs in different blood and solid tumors [183], most recently in infant ALL [184]. The concept of bi- and tri-specific antibodies has been used to target NK cell activity, leading to the rise of bi- and tri-specific NK cell engagers.

Several NK cell engagers have been investigated in clinical trials, both as a monotherapy or in combination with other therapies, in both hematological and solid malignancies [185]. Several platforms have emerged to harness NK cell-mediated antibody-dependent cellular cytotoxicity through CD16 [185,186] and NKp46 [187,188] targeting, with some also being explored in combination with PD-1 blockade [185,189]. Bi-specific and tri-specific killer engagers (BiKE and TriKE molecules, respectively) also engage CD16 and tumor targets, with TriKEs the first to also incorporate an IL-15 moiety, delivering additional increased NK cell proliferation and survival signals [185]. Second-generation TriKEs have been developed, with modifications to incorporate wildtype IL-15 to improve NK-specific proliferation and tumor control [190]. cam1615B7H3 is a novel second-generation TriKE that targets B7-H3-expressing tumors. It has shown promising therapeutic efficacy against solid tumors, both in vitro and in vivo, while delivering IL-15 signals more specifically to NK cells [191]. The combination of NK-mediated ADCC and cytokine signaling using TriKEs is a promising approach to clinical NK cell therapies, leading to a first-generation TriKE, GTB-3550 (CD16/IL-15/CD33) being investigated against CD33^+^ hematological malignancies (NCT03214666).

### 5.5. Potential of ILC-Based Immunotherapies in Melanoma

Due to their enrichment in the skin and their roles in maintaining homeostasis and tissue repair, ILCs present a clear opportunity for initiating early immune responses against cancerization [192,193]. For one, NK cell fluctuations in both frequency and phenotype can be used as theragnostic biomarkers to predict response in melanoma therapy [192]. Metastatic melanoma patients that respond to anti-PD-1 therapy show an increase in density of intratumoral NK cells, which when compared to non-responders, are also in closer proximity to melanoma cells [194]. Furthermore, Lee et al. suggest that patients with low expression of MHC class I, but high NK density, may continue to respond to anti-PD-1 therapy through the cooperative recognition of MHC class I low cells by NK cells and MHC class I-expressing cells by CD8^+^ T cells. Although they have been shown to infiltrate melanoma, NK cell-based therapies are not very potent toward primary solid tumors, and melanoma cells can utilize different mechanisms to evade NK cell-mediated lysis [192,195]. Autologous and allogenic NK cell therapies have been studied in the clinic against melanoma, but their efficacy remains limited. Vliet et al. recently explored the barriers to improving efficacy, such as better NK cell tumor infiltration, and the combination of strategies to manipulate the TME and immune system, boosting the impact of adoptive NK cell therapy [196]. Interestingly, chemotherapeutics used to treat metastatic melanoma have the potential to support and enhance NK cell function in the TME. Therefore, the combination of NK-based immunotherapies and improved nanotechnology-based chemotherapeutic delivery presents another promising approach for melanoma treatment [197].

## 6. Conclusions

ILCs form a complex and heterogenous family of innate immune cells that have shown a promising capacity to be targeted in cancer. Immune checkpoint inhibition is a field currently explored in ILCs, with growing clinical potential. Although the last 15 years have resulted in considerable discoveries in ILC biology, there is much to uncover concerning their incredible diversity, their ability to transdifferentiate, and the tissue-specificity of their responses in cancer. While in their infancy, the concepts discussed here to harness the activity of ILCs beyond ICB—targeting cytokines, modulating ILC phenotype, engineering CAR and TCR ILCs, and developing NK cell engager vectors—are all potential pathways that warrant further investigations. Some key challenges that these approaches must resolve are improving the targeting accuracy and localization to reduce off-target effects and ensuring that the subsequent anti-tumor ILC function in the TME is both strong and durable. Either alone or in combination with innate or adaptive strategies, the development of unique ILC-mediated therapies can bring forth new ways to improve efficacy as well as to overcome resistances to the current standard of care in melanoma and other cancers, with the ultimate goal of improving patient outcomes.

## Figures and Tables

**Table 1:** FDA-Approved Immune Checkpoint Inhibitors.

Antibody	Target	FDA Approval with Initial Indication	Subsequent Indications	References
ipilimumab	CTLA-4	2011 for unresectable or metastatic melanoma	melanoma (alone or w/nivolumab) other solid tumors (w/nivolumab)	[77,78]
tremelimumab	CTLA-4	2022 for unresectable HCC (w/durvalumab), and metastatic NSCLC (w/durvalumab and platinum-based chemotherapy)		[79]
pembrolizumab	PD-1	2014 (accelerated) for unresectable or metastatic melanoma 2015 (accelerated) for metastatic NSCLC	many solid tumors cHL, PMBCL	[78,80,81]
nivolumab	PD-1	2014 (accelerated) for unresectable or metastatic melanoma 2015 for advanced RCC and metastatic squamous and non-squamous NSCLC 2016 (accelerated) for relapsed or progressive cHL	many solid tumors	[78,82,83,84,85]
cemiplimab	PD-1	2018 for locally advanced or metastatic CSCC	BCC, NSCLC	[86,87,88]
dostarlimab	PD-1	2021 (accelerated) for dMMR recurrent or advanced endometrial cancer		[89]
atezolizumab	PD-L1	2016 (accelerated) for locally advanced or metastatic urothelial carcinoma 2016 for metastatic NSCLC	other solid tumors	[78,90,91]
avelumab	PD-L1	2017 (accelerated) for metastatic MCC and locally advanced or metastatic urothelial carcinoma	RCC	[78,92,93]
durvalumab	PD-L1	2017 (accelerated) for locally advanced or metastatic urothelial carcinoma	NSCLC, SCLC, BTC	[78,94]
relatlimab	LAG-3	2022 for unresectable or metastatic melanoma (w/nivolumab, market name Opdualag)		[95]

BCC, basal cell carcinoma; BTC, biliary tract cancer; cHL, classical Hodgkin’s lymphoma; CSCC, cutaneous squamous cell carcinoma; CTLA-4, cytotoxic T-lymphocyte antigen 4; dMMR, deficient mismatch repair; HCC, hepatocellular carcinoma; LAG-3, lymphocyte activation gene-3; MCC, Merkel cell carcinoma; NSCLC, non-small cell lung cancer; PD-1, programmed cell death protein 1; PD-L1, programmed death-ligand 1; PMBCL, primary mediastinal B-cell lymphoma; RCC, renal cell carcinoma; SCLC, small cell lung cancer; w/, with.

## Data Availability

No new data were created or analyzed in this study. Data sharing is not applicable to this article.
